# An empirical model of carbon-ion relative biological effectiveness based on the linear correlation between radiosensitivity to photons and carbon ions

**DOI:** 10.1088/1361-6560/ad918e

**Published:** 2024-12-10

**Authors:** David B Flint, Scott J Bright, Conor McFadden, Teruaki Konishi, David K J Martinus, Mandira Manandhar, Mariam Ben Kacem, Lawrence Bronk, Gabriel O Sawakuchi

**Affiliations:** 1 Department of Radiation Physics, The University of Texas MD Anderson Cancer Center, Houston, TX, United States of America; 2 Department of Radiation Regulatory Science Research, Institute for Radiological Science, National Institutes for Quantum Science and Technology, Inage-ku, Chiba, Japan; 3 The University of Texas MD Anderson Cancer Center UTHealth Houston Graduate School of Biomedical Sciences, Houston, TX, United States of America

**Keywords:** particle therapy, RBE, linear energy transfer, radiosensitivity, modeling, carbon ion

## Abstract

*Objective.* To develop an empirical model to predict carbon ion (C-ion) relative biological effectiveness (RBE). *Approach.* We used published cell survival data comprising 360 cell line/energy combinations to characterize the linear energy transfer (LET) dependence of cell radiosensitivity parameters describing the dose required to achieve a given survival level, e.g. 5% (D_5%_), which are linearly correlated between photon and C-ion radiations. Based on the LET response of the metrics D_5%_ and D_37%_, we constructed a model containing four free parameters that predicts cells’ linear quadratic model (LQM) survival curve parameters for C-ions, *α*_C_ and *β*_C_, from the reference LQM parameters for photons, *α_X_* and *β_X_*, for a given C-ion LET value. We fit our model’s free parameters to the training dataset and assessed its accuracy via leave-one out cross-validation. We further compared our model to the local effect model (LEM) and the microdosimetric kinetic model (MKM) by comparing its predictions against published predictions made with those models for clinically relevant LET values in the range of 23–107 keV *μ*m^−1^. *Main Results.* Our model predicted C-ion RBE within ±7%–15% depending on cell line and dose which was comparable to LEM and MKM for the same conditions. *Significance.* Our model offers comparable accuracy to the LEM or MKM but requires fewer input parameters and is less computationally expensive and whose implementation is so simple we provide it coded into a spreadsheet. Thus, our model can serve as a pragmatic alternative to these mechanistic models in cases where cell-specific input parameters cannot be obtained, the models cannot be implemented, or for which their computational efficiency is paramount.

## 1. Introduction

Particle therapy with carbon ions (C-ions) has several benefits over photon and proton beam therapies, including inherently superior depth-dose distributions (Tsujii *et al* 2008, Kamada *et al* 2015), less dependence on tumor oxygenation status (Nakano *et al* 2006, Tinganelli and Durante 2020), and increased relative biological effectiveness (RBE) (Tsujii *et al* 2008, Kamada *et al* 2015). However, as RBE depends strongly on the linear energy transfer (LET) and dose (Karger and Peschke 2017, Stewart *et al* 2018), both of which vary greatly across clinical treatment fields, variable C-ion RBE models are needed in clinical scenarios to account for the differential cell killing at different depths in a treatment plan (Karger and Peschke 2017, Tinganelli and Durante 2020). Several mechanistic models are currently in use to account for these differences in clinical scenarios, notably: the microdosimetric kinetic model (MKM) (Hawkins 1994, 2003) explains the variable RBE in terms of changes in microdosimetric quantities such as dose-mean lineal energy; and the local effect model (LEM) (Scholz and Kraft 1994, Scholz *et al* 1997, Weyrather *et al* 1999) explains the variable RBE in terms of microscopic dose heterogeneity. While a number of other mechanistic models have been proposed to account for biophysical considerations that drive cell response to heavy ions, including the induction of complex DNA damage (Carante *et al* 2019) and oxidative stress (Cunha *et al* 2017), currently only the MKM and LEM have been implemented clinically.

Unlike RBE modeling efforts for protons, where a comparatively large number of empirical models have also been proposed (Carabe *et al* 2012, Wedenberg *et al* 2013, McNamara *et al* 2015, Mairani *et al* 2017, Flint *et al* 2022), C-ion RBE modeling has been focused primarily on mechanistic model development, with empirical models rarely being considered against their mechanistic counterparts. This is a missed opportunity because, while empirical models are typically not very informative of the underlying mechanisms driving the response, they can nevertheless be very useful owing to the relatively few input parameters they require and their very minimal computational expense which greatly simplifies both their implementation and use. For example, while in principle, an empirical model may only require three input parameters (the reference cell survival curve parameters *α* and *β* from the linear quadratic model [LQM] and a beam quality specifier such as the LET), mechanistic models tend to be much more sophisticated, both in terms of the number of input parameters required and the complexity of obtaining them. For instance, the MKM model requires six input parameters (the dose-mean lineal energy, the subnuclear domain size, nuclear cross-section for lethal lesions, the linear-quadratic model parameter limit, reference alpha/beta ratio, and the RBE in the limit as the lineal energy approaches zero), while the LEM requires five input parameters (beam energy spectrum, LET spectrum, reference *α*, reference *β*, dose threshold) (Stewart *et al* 2018). If these values are not available for a given cell line, then they must be approximated by representative values, which is not ideal if one is trying to leverage any additional accuracy these mechanistic models might confer.

This issue is exacerbated by the fact that the additional cell-line specific parameters required by mechanistic models are rarely provided alongside the published survival data and determining them is not trivial; requiring access to a biological wetlab to culture the cells, specialized equipment to make the measurements, and the expertise needed to perform these tasks. Also, the sophisticated beam-quality parameters needed by these mechanistic models are similarly difficult to obtain, being rarely reported alongside survival data, and must be computed via Monte Carlo simulations requiring a validated model of the specific beamline to score the required quantities with confidence. Thus, a broad, fair validation of these mechanistic models against the vast collection of survival data in the literature is very challenging owing to the lack of the input parameters needed to make the calculations besides the few cell lines and beam lines for which these parameters have been determined. This is not an issue, however, for the simpler empirical models, because survival studies routinely report the reference *α* and *β* values alongside beam quality specifiers such as the dose- (or fluence-) weighted LET. Indeed, such studies benchmarking the response of empirical models in the context of proton RBE have been recently published (Gardner *et al* 2024), owing largely to the fact that these empirical models can very easily be implemented and used to make predictions against data in the literature.

While recent developments in machine learning have led to the creation of several AI-based RBE models (Papakonstantinou *et al* 2021, Cordoni *et al* 2023) that fall broadly into the category of empirical data-driven approaches, these machine learning approaches share many of the same practical limitations as the mechanistic models, being challenging and computationally expensive to implement, and requiring a great number of input parameters that may not be readily available when trying to make predictions on new data. Thus, we sought to develop an empirical C-ion RBE model that is easier to implement and use than its mechanistic counterparts, and which in turn could be easily benchmarked against published predictions made with these models. Towards this, we developed our model based on the linear correlations between radiosensitivity to photons and C-ions observed by Suzuki *et al* (2000), and which we previously showed could be used as the basis of a proton RBE model (Flint *et al* 2022). This new C-ion RBE model contains only four free parameters and requires only three inputs: the dose-weighted LET (LET*_d_*) and a cell line’s reference photon *α_x_* and *β_x_* values. We further show that our model has comparable accuracy to the LEM and MKM models across clinically relevant C-ion LET values, despite requiring the fewest input parameters. Thus, our model could be used as a pragmatic tool to rapidly and accurately assess RBE-weighted C-ion doses in scenarios where the input parameters for the LEM or MKM cannot be obtained. To underscore the utility of our model and the simplicity of our approach, as supplemental material we provide an implementation of our model in an Excel spreadsheet as well as a MATLAB function that can rapidly predict C-ion cell survival curves at any clinically relevant LET for any cell line whose *α_x_* and *β_x_* values are known.

## 2. Methods and materials

### 2.1. Assessment of linear correlations between photon and C-ion radiosensitivity

To assess the linear correlations between photon and C-ion radiosensitivity, we analyzed previously published cell survival data for eight human cancer cell lines (H460 and H1299 non-small cell lung cancer; BxPC3, PANC-1, AsPC1, and Panc 10.05 pancreatic adenocarcinoma; and M059K and M059J glioblastoma cells) exposed to C-ions with LET*_d_* values of 13.5, 27.9, and 60.5 keV *μ*m^−1^, with 6 MV x-rays as a reference radiation source (Flint *et al* 2021a, 2021b). We extracted the radiosensitivity parameters D_5%_, D_10%_, D_14%_, D_20%_, D_37%_, and D_50%_ (the dose required to achieve 5%, 10%, 14%, 20%, 37% and 50% survival respectively) from the survival curves and determined the correlation between radiation qualities via the Pearson correlation coefficient (*r*). These data were selected for this assessment as they represent: (1) a large cohort of cell lines (*N* = 8) spanning a wide range of intrinsic radio sensitivities (D_10%,*x*_ = [1.37 ± 0.02, 8.56 ± 0.19]), allowing the linear trends to be readily observable; (2) data collected by the same authors under the same experimental conditions to reduce uncertainties due to experimental setup; and (3) data spanning a reasonable range of clinically relevant LET values.

### 2.2. Training dataset

To establish a dataset to train our model, we combined the C-ion data summarized in the PIDE Database version 3.2 (Friedrich *et al* 2013, 2021), data from our previous C-ion studies (Flint *et al* 2021a, 2021b) and data from Mein *et al*’s study comparing the predictions of the LEM and MKM models (Mein *et al* 2020). These data comprised a total of 433 cell line/LET combinations. From this database, we removed data according to the following criteria: data where the reference photon source energy was less than 200 kVp (*n* = 21 datasets) to ensure that very low energy x-ray sources that may have RBE > 1 relative to high energy x-ray (or gamma ray) sources did not perturb the trends we characterized; data with LET > 350 keV *μ*m^−1^ (*n* = 15 datasets) to ensure our model was trained on clinically relevant LET values, and because survival data acquired under such high LET conditions have considerably larger dosimetric uncertainties; and data where the LQM survival curve parameter *β* was reported as negative (*n* = 44 datasets), because negative *β* values can occasionally cause endpoints such as D_10%_ to have complex values, which negatively affects the performance of the fitting algorithms used to find the model’s free parameters. This resulted in the inclusion of a total of 360 cell line/LET combinations (83% of the available data). All LET values reported in this work are dose-weighted LET*_d_* values.

### 2.3. Establishing underlying trends

Similar to our approach for protons (Flint *et al* 2022), we selected several candidate functions to assess how the slope and intercept of the linear correlations between radiosensitivity metrics varied with LET*_d_*, combining them into a single function describing the LET response for a given endpoint, e.g. for D_10%_:

\begin{equation*}{{\text{D}}_{10\% ,C}} = { }{{\text{D}}_{10\% ,X}} \cdot {\text{Slope}}{\left( {{\text{LE}}{{\text{T}}_d}} \right)_i} + {\text{Intercept}}{\left( {{\text{LE}}{{\text{T}}_d}} \right)_i}.\end{equation*}

This allowed us to assess how well different functions describe the training data without the need for binning the data into like-LET bins. Given our previous findings that proton RBE data were best described by minimally parameterized functions (Flint *et al* 2022), we constrained our search to functions of three or fewer parameters for both the slope and intercept. We assessed 23 candidate functions for the slope, and 15 candidate functions for the intercept (listed in supplementary note S1), and, for every pair thereof, we determined the values of the combined functions’ free parameters by minimizing the square relative distance between the measured and predicted endpoints. We then calculated the Bayesian information criteria (BIC) associated with each function’s predictions of that endpoint across the training dataset to identify the candidate functions that best described how these radiosensitivity metrics vary with C-ion LET*_d_* without the inclusion of unwarranted free parameters in that description.

The BIC was chosen as the goodness-of-fit metric when establishing the trends because it uses a larger penalty for the inclusion of free parameters than the Aikake information criteria (AIC). Since our formalism would ultimately require predictions across multiple endpoints to be incorporated into our final model, we sought to minimize the number of free parameters used in each endpoint’s description to prevent our model from becoming overly complex once multiple endpoints were incorporated.

### 2.4. Choice of endpoints to incorporate into our model

As we showed previously, with a set of functions similar to equation (1) but for several radiosensitivity endpoints, a model can be constructed that predicts *α_C_* and *β_C_* from *α_X_* and *β_X_* in a manner analgous to fitting the set of predicted endpoints to the LQM (Flint *et al* 2022). However, it is not immediately obvious how many such functions suffice to predict the response. To determine this for C-ions, we selected the best performing functions for the endpoints (*e*^−3^ ≈ 5% survival), ${D_{{e^{ - 2}}}}$ (*e*^−2^ ≈ 14% survival), ${D_{{e^{ - 1}}}}$ (*e*^−1^ ≈ 37% survival), ${D_{{e^{ - 1/2}}}}$ (*e*^−1/2^ ≈ 61% survival), and, for each 2-, 3-, and 4-endpoint combination of functions, we predicted the C-ion survival curves across the training data according to the expressions given in our previous work (Flint *et al* 2022):

\begin{equation*}{\alpha _{{\text{proton}}}} = { }\frac{{{{\mathop \sum \nolimits}}_iD_{{\text{SF}},i}^4{{\mathop \sum \nolimits}}_{\boldsymbol{i}}{D_{{\text{SF}},i}}\log \left( {{\text{S}}{{\text{F}}_i}} \right) - {{\mathop \sum \nolimits}}_i{ }D_{{\text{SF}},i}^3{{\mathop \sum \nolimits}}_{\boldsymbol{i}}D_{{\text{SF}},i}^2\log \left( {{\text{S}}{{\text{F}}_i}} \right)}}{{{{\mathop \sum \nolimits}}_iD_{{\text{SF}},i}^3{{\mathop \sum \nolimits}}_i{ }D_{{\text{SF}},i}^3 - {{\mathop \sum \nolimits}}_iD_{{\text{SF}},i}^2{ }{{\mathop \sum \nolimits}}_iD_{{\text{SF}},i}^4}}{ }\end{equation*}

and

\begin{equation*}{\beta _{{\text{proton}}}} = { }\frac{{{{\mathop \sum \nolimits}}_iD_{{\text{SF}},i}^2{{\mathop \sum \nolimits}}{_{\boldsymbol{i}}}D_{{\text{SF}},i}^2\log \left( {{\text{S}}{{\text{F}}_i}} \right) - {{\mathop \sum \nolimits}}_iD_{{\text{SF}},i}^3{{\mathop \sum \nolimits}}{_{\boldsymbol{i}}}{D_{{\text{SF}},i}}\log \left( {{\text{S}}{{\text{F}}_i}} \right)}}{{{{\mathop \sum \nolimits}}_iD_{{\text{SF}},i}^3{{\mathop \sum \nolimits}}_iD_{{\text{SF}},i}^3 - {{\mathop \sum \nolimits}}{_i}D_{{\text{SF}},i}^2{ }{{\mathop \sum \nolimits}}_iD_{{\text{SF}},i}^4}}.\end{equation*}

We then calculated the corrected AIC (AICc) associated with each differently constructed model to identify how many and which endpoints were sufficient to predict the survival curves. Here, the distance metric we used was the L2 norm, defined by integrating the squared distance between the predicted and measured curves over the dose range [0.5, 4] Gy, normalized by the integral of the predicted curve over this range:

\begin{equation*}L{2_{0.5 - 4\,{\text{Gy}}}} = \frac{{\sqrt {{{\mathop \int \nolimits}}_{0.5{\text{ Gy}}}^{4{\text{ Gy}}}{{\left( {{\text{S}}{{\text{F}}_{{\text{measured}}}}\left( D \right) - {\text{ S}}{{\text{F}}_{{\text{predicted}}}}\left( D \right)} \right)}^2}{\text{d}}D} }}{{{{\mathop \int \nolimits}}_{0.5{\text{ Gy}}}^{4{\text{ Gy}}}{\text{S}}{{\text{F}}_{{\text{predicted}}}}\left( D \right){\text{d}}D}}.\end{equation*}

This distance metric was used because it minimizes the relative area between the predicted and measured curves across clinically relevant dose levels. This is a small departure from our previous approach, where the integral across all dose levels was considered (Flint *et al* 2022). Here, for C-ion survival curves, we found that owing to the rapidly decreasing nature of the survival curves for higher LET ions, integrating the entire dose range strongly weighs the very-low-dose portion of the curve, where survival is still relatively high. The biological uncertainties in these regions are quite high, and so constraining our dose range in this way avoids biasing our fits towards data in which we have less confidence.

### 2.5. Determining the free parameter values for our model

Once we established which radiosensitivity endpoints to incorporate into our model, we determined the free parameter values by fitting the final function to the training dataset, minimizing the sum-of-square L2_0.5−4G*y*_ values between the predicted and measured curves via MATLAB 2020’s *fmincon* function (Mathworks, Natick, MA). Because using this distance metric tends to result in fitting that is sensitive to the initial choice parameters, we randomly generated 10 000 initial guesses within ±10% of the values determined for each endpoint alone, minimized the sum-of-square L2_0.5−4G*y*_ values using *fmincon*, and then selected the parameterization that resulted in the best overall description of the data.

### 2.6. Assessing model accuracy

To quantify the accuracy of our model, we performed leave-one-out cross-validation, retraining the model after holding out each point, and using the new parameterization to predict the held-out point. This method was chosen as the training data are distributed unevenly across cell lines and LET values, and leave-one-out cross-validation provides an approximately unbiased estimate of the prediction uncertainty even across unbalanced datasets (Berrar 2019). We calculated the relative deviations between the predicted and measured RBE values at the 0.5, 1, 2, and 4 Gy dose levels and bootstrapped them to estimate prediction intervals associated with our model. To further assess how our model’s accuracy varied with C-ion LET, we grouped the training data by C-ion LET*_d_* values roughly by where these LET values occur in the context of clinical spread-out Bragg peaks (SOBPs), and assessed the accuracy in each region independently. For the predictions shown in §3.5–3.7, the data shown are those predicted during the leave-one-out cross-validation step, such that the model predicting each datapoint was not trained on that datapoint.

### 2.7. Statistical analyses

We fit the free parameters of our model and assessed our model’s performance in MATLAB 2020, while the correlations between the radiosensitivity metrics were quantified in Graph Pad Prism 9 (Graph Pad Software, San Diego, CA). The confidence intervals in the measured survival curves were calculated by propagating the standard error in the fit of *α* and *β* to the LQM, along with their covariances, to estimate the surviving fraction according to the LQM. The confidence intervals in the predicted survival curves were calculated after propagating the uncertainty in the model’s parameter values, along with their covariances, through our model. The confidence intervals given are ±1.96 times the standard error of the predictions, which are approximately 95% confidence intervals.

## 3. Results

### 3.1. C-ion radiosensitivity parameters correlate with photon radiosensitivity parameters regardless of choice of endpoint

**Figure 1.**
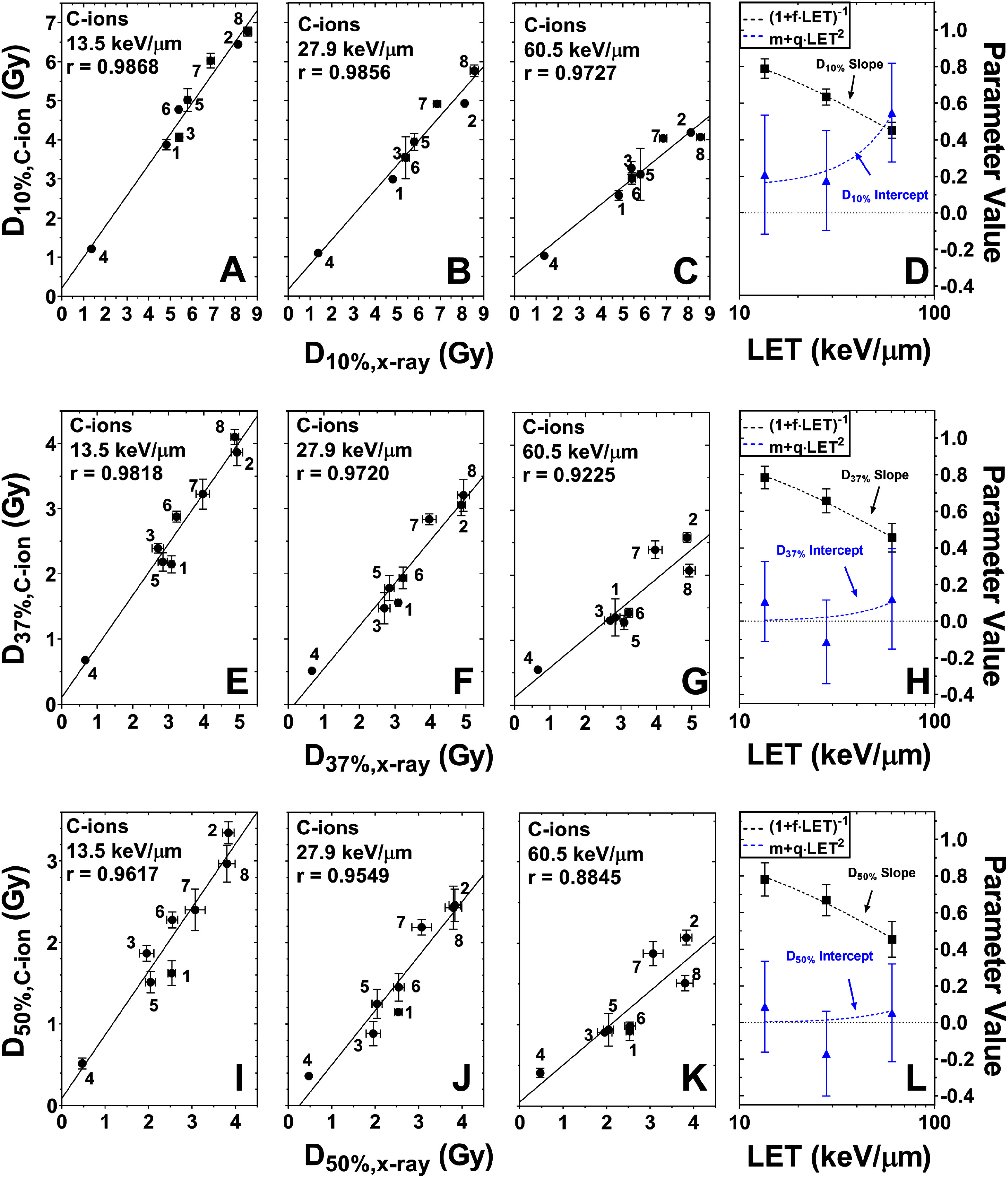
Linear correlation between C-ion and 6-MV x-ray radiosensitivity for the endpoints D_10%_ (A)–(C); D_37%_ (E)–(G); and D_50%_ (I)–(K), for cells exposed to C-ions with LET_*d*_ values of 13.5 keV *μ*m^−1^ (A), (E), (I), 27.9 keV *μ*m^−1^ (B), (F), (J) or 60.5 keV *μ*m^−1^ (C), (G), (K) and 6 MV x-rays as a reference source. Values for the slopes (black) and intercepts (blue) of the correlations are given in panels *D*, *H* and *L*, with the dashed lines showing fits of the data. Numbers within the panels indicate the cell lines: 1 = H460, 2 = H1299, 3 = M059K, 4 = M059J, 5 = BxPC3, 6 = AsPC1, 7 = PANC-1, and 8 = Panc 10.05. Results for other endpoints can be found in supplementary note S2.

As first shown by Suzuki *et al* (2000), the radiosensitivity endpoint D_10%_ is linearly correlated between photon and C-ion radiation. Our analyses (figures 1(A)–(C)) confirm this result and also our previous results for protons (Flint *et al* 2022) that these correlations hold regardless of the choice of radiosensitivity metric (figures 1(E)–(G) and (I)–(K)). As is true for protons, the slopes of these correlations decrease with LET*_d_*, although the behavior of the intercept is less clear owing to the uncertainties in estimating them (figures 1(D), (H) and (L)).

### 3.2. Choice of slope and intercept parameterizations for different endpoints

When we assessed the predictive power of different parameterizations to predict each radiosensitivity endpoint, we found that several parameterizations offered comparable descriptions of the data (see Supplementary Note S1). For lower survival levels, the LET*_d_* dependence of the slope tended to be best described by an inverse linear function with one free parameter, *f*:

\begin{equation*}{\text{Slope}}\left( {{\text{LE}}{{\text{T}}_d}} \right) = {\left( {1 + f \cdot {\text{LE}}{{\text{T}}_d}} \right)^{ - 1}}\end{equation*}

whereas for higher survival levels, the slope was best described by the square of an inverse linear function:

\begin{equation*}{\text{Slope}}\left( {{\text{LE}}{{\text{T}}_d}} \right) = {\left( {1 + f \cdot {\text{LE}}{{\text{T}}_d}} \right)^{ - 2}}.\end{equation*}

Regardless of survival level, the intercept’s LET*_d_* dependence was generally best described by a purely quadratic function with a small vertical offset, containing two free parameters (*q* and *m*):

\begin{equation*}{\text{Intercept}}\left( {{\text{LE}}{{\text{T}}_d}} \right) = q \cdot {\text{LE}}{{\text{T}}_d}^2 + m.\end{equation*}

### 3.3. Choice of endpoints incorporated into our model

As we previously showed that using endpoints with survival levels corresponding to powers of *e* (e.g. *e*^−3^ ≈ 5%) simplifies the final model formulation (Flint *et al* 2022), we investigated the endpoints ${D_{{e^{ - 3}}}}$, ${D_{{e^{ - 2}}}}$ ${D_{{e^{ - 1}}}}$, and ${D_{{e^{ - 1/2}}}}$, choosing the function best describing each endpoint, and, for every 2-, 3-, and 4-endpoint combination thereof, we evaluated the ability of the resulting model (via equations (2) and (3)) to predict the response. Like our previous results for protons, our data here suggest that incorporating only two endpoints is sufficient to predict the response for C-ions (table 1).

**Table 1.** Corrected Aikake information criteria (AICc) values of models constructed with different combinations of endpoints. Shown are AICc values for the best combination (minimum AICc) when including a given number of endpoints.

Endpoints used	2	3	4
Minimum AICc value Across combinations	–163.4	–156.3	–147.4

Among the two-endpoint combinations, (${D_{{e^{ - 3}}}}$, ${D_{{e^{ - 2}}}}$) and (${D_{{e^{ - 3}}}}$, ${D_{{e^{ - 1}}}}$) provided the best overall descriptions of the data (table 2). As $D_{e^{-3}}$ and $D_{e^{-2}}$ correspond to relatively low survival levels (∼5% and ∼14%, respectively), we chose to incorporate the endpoints ${D_{{e^{ - 3}}}}$ and ${D_{{e^{ - 1}}}}$ into the construction of our model, as this pair provides a similarly good description of the data as ${D_{{e^{ - 3}}}}$ and ${D_{{e^{ - 2}}}}$ while avoiding introducing any potential bias towards higher dose/lower survival levels.

**Table 2.** Corrected Aikake information criteria (AICc) values for models constructed using two endpoints, showing the differences in goodness-of-fit obtained after selecting different endpoints.

AICc values			
Endpoint pair	${D_{{e^{ - 2}}}}$	${D_{{e^{ - 1}}}}$	${D_{{e^{ - 1/2}}}}$
${{{D}}_{{{{e}}^{ - 3}}}}$	–163.4	–162.6	–155.4
${{{D}}_{{{{e}}^{ - 2}}}}$		–144.6	–135.7
${{{D}}_{{{{e}}^{ - 1}}}}$			–56.7

### 3.4. C-ion model parameterization

Incorporating ${D_{{e^{ - 3}}}}$ and ${D_{{e^{ - 1}}}}$ as endpoints simplifies the resulting parameterization of equations (2) and (3) as follows (see supplementary note S3 for details):

\begin{equation*}{\alpha _C} = \,\frac{{D_{{e^{ - 3}}}^2 - 3\,D_{{e^{ - 1}}}^2}}{{{D_{{e^{ - 1}}}}D_{{e^{ - 3}}}^2 - {D_{{e^{ - 3}}}}D_{{e^{ - 1}}}^2}}\,\end{equation*}

and

\begin{equation*}{\beta _C} = \,\frac{{3\,{D_{{e^{ - 1}}}} - \,{D_{{e^{ - 3}}}}}}{{{D_{{e^{ - 1}}}}D_{{e^{ - 3}}}^2 - {D_{{e^{ - 3}}}}D_{{e^{ - 1}}}^2}}\,.\end{equation*}

Similar to our previous work (Flint *et al* 2022), when alpha or beta are found to be negative by equations (8) and (9), we constrained them to be non-negative by setting either parameter to zero and solving for the other parameter alone as follows (see for supplementary note S3 details):

\begin{equation*}{\alpha _{C\,\left( {\beta = 0} \right)}} = \,\frac{{3\,{D_{{e^{ - 3}}}} + \,{D_{{e^{ - 1}}}}}}{{D_{{e^{ - 3}}}^2 + D_{{e^{ - 1}}}^2}}\,\end{equation*}

and

\begin{equation*}{\beta _{C{ }\left( {\alpha = 0} \right)}} =\frac{{3{ }D_{{e^{ - 3}}}^2 + { }D_{{e^{ - 1}}}^2}}{{D_{{e^{ - 3}}}^4 + D_{{e^{ - 1}}}^4}}{ }.\end{equation*}

To determine the expressions used for ${D_{{e^{ - 3}}}}$ and ${D_{{e^{ - 1}}}}$ in equations (8)–(11), we used equations (5)–(7) to train models for two endpoints (SF_1_ = *e*^−3^ and SF_2_ = *e*^−1^) on the training data and minimized the AICc value associated with the L2_0.5−4 Gy_ when globally fitting the free parameters.

This fitting procedure yielded values of *q* and m for SF_2_ = *e*^−1^ that were approximately zero, suggesting that the deviation of the intercept from zero at this survival level is not sufficient to warrant the use of any free parameters to describe its behavior. Accordingly, we refit our model, omitting the intercept term from the description of ${D_{{e^{ - 1}}}},$ and found that this indeed yielded superior AICc values for the resulting model (supplementary note S4). Finally, we refit the model but using equation (5) to describe ${D_{{e^{ - 1}}}}$, because we found when modeling the endpoints themselves (supplementary note S1), that when the intercept term is omitted, an inverse linear function similar to equation (5) provides a better description of the endpoints than the square of an inverse linear function similar to equation (6). This description minimized the AICc associated with the model’s predictions (supplementary note S4), and the final expressions used for ${D_{{e^{ - 3}}}}$ and ${D_{{e^{ - 1}}}}$ in our model were therefore:

\begin{equation*}{D_{{e^{ - 3}},C}} = { }{D_{{e^{ - 3}},X}} \cdot {\left( {1 + {f_1} \cdot {\text{LE}}{{\text{T}}_d}} \right)^{ - 1}} + {q_1} \cdot {\text{LE}}{{\text{T}}_d}^2 + {m_1}\end{equation*}

and

\begin{equation*}{D_{{e^{ - 1}},C}} = { }{D_{{e^{ - 1}},X}} \cdot {\left( {1 + {f_2} \cdot {\text{LE}}{{\text{T}}_d}} \right)^{ - 1}}\end{equation*}

with the free parameter values summarized in table 3.

**Table 3.** Free parameter values for our model, their uncertainties, and their covariance. Dose units are assumed to be in Gy, and LET_*d*_ units in keV *μ*m^−1^.

Parameter	Units	Value	Uncertainty
f_1_	(keV/*μ*m)^−1^	2.095 070 × 10^−2^	8.575 542 × 10^−3^
m_1_	Gy	5.955 453 × 10^−1^	4.089 380 × 10^−1^
q_1_	(keV/*μ*m)^−2^	2.133 918 × 10^−5^	8.756 112 × 10^−6^
f_2_	(keV/*μ*m)^−1^	2.230 798 × 10^−2^	3.479 508 × 10^−3^
cov_f1,m1_	Gy (keV/*μ*m)^−1^	2.945 233 × 10^−3^	N/A
cov_f1,q1_	(keV/*μ*m)^−3^	4.704 403 × 10^−9^	N/A
cov_f1,f2_	(keV/*μ*m)^−2^	1.172 688 × 10^−6^	N/A
cov_q1,m1_	Gy (keV/*μ*m)^−2^	–8.767 703 × 10^−7^	N/A
cov_m1,f2_	(keV/*μ*m)^−1^	–6.539 078 × 10^−6^	N/A
cov_q1,f2_	(keV/*μ*m)^−3^	1.257 442 × 10^−9^	N/A

### 3.5. Prediction of cell survival curves

**Figure 2.**
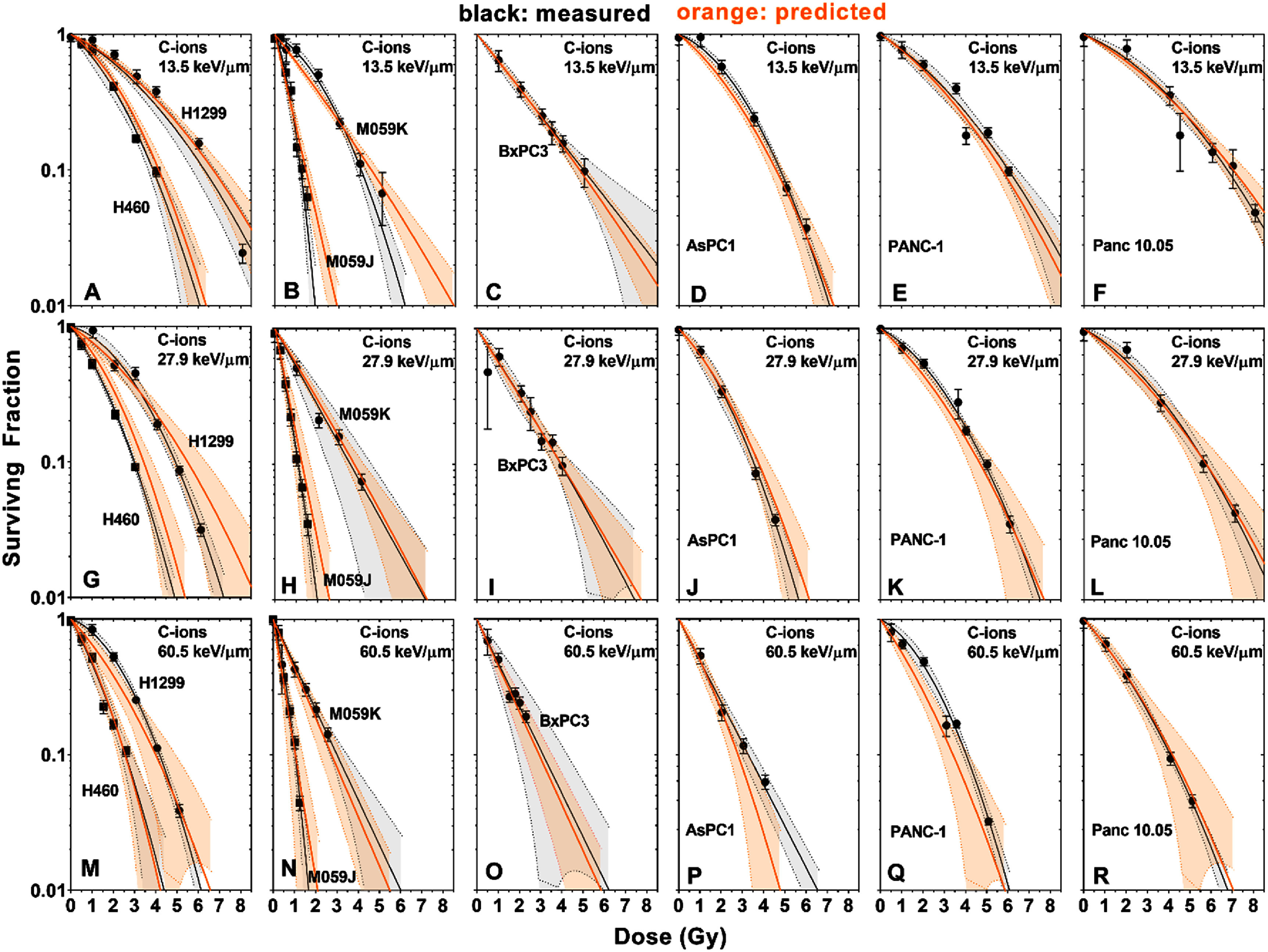
Measured cell survival curves (Flint *et al* 2021b) (black) and our model’s predictions of the same data (orange), for cells exposed to C-ions with LET values of 13.5 (A)–(F), 27.9 (G)–(L), and 60.5 keV *μ*m^−1^ (M)–(R). (A), (G), (M) H460 and H1299 cells; (B), (H, (N) M059K and M059J cells; (C), (I), (O) BxPC3 cells; (D), (J), (P) AsPC1 cells; (E), (K), (Q) PANC-1 cells; (F), (L), (R) Panc 10.05 cells. Grey and orange bands show 95% confidence intervals around the measured or predicted survival curves, respectively.

Our model predicts survival curves via equations (8)–(13), requiring only *α_X_, β_X_*, and the LET*_d_* as inputs. To demonstrate this, we used our model to predict the response of the eight cell lines whose survival was reported in our previous C-ion studies (Flint *et al* 2021a, 2021b). These predictions, shown in figure 2, generally show very good agreement between our model and the measured survival curves, with the measured and predicted curves consistently falling within each other’s confidence bands. Notably, these predictions were made with the model parameters obtained during the leave-one-out cross-validation step (as described in § 2.6) so as not to bias the predictions by including the predicted data in the training dataset.

### 3.6. Reproducing literature RBE trends

**Figure 3.**
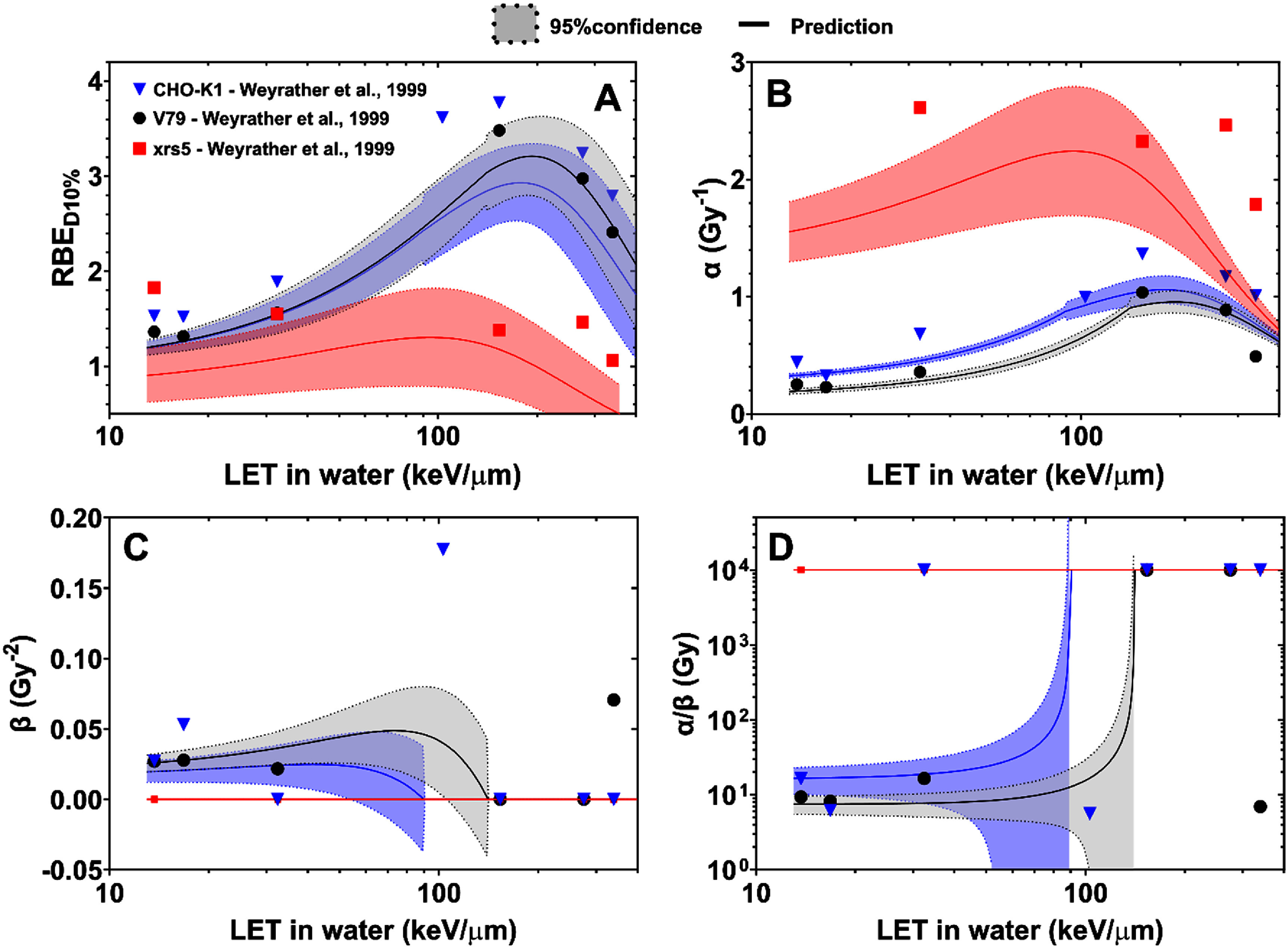
Predictions of the LET_*d*_ response of (A) the RBE at the 10% survival level, (B) the survival curve parameter *α*, (C) the survival curve parameter *β*, and (D) the ratio *α/β* for the data measured by Weyrather *et al* (1999) (as reported in the PIDE) for cells of greatly varying radiosensitivities: V79 (black circles, D_10%_ = 7.8 ± 0.7 Gy), CHO-K1 (blue triangles, D_10%_ = 6.45 ± 0.3 Gy), and xrs5 (red squares, D_10%_ = 1.34 ± 0.07 Gy). The points show the measured data, the solid lines represent the predictions of our model and the dashed lines represent 95% confidence intervals around the predictions based on the uncertainties in the model parameters. In panel (*D*), divergent *α/β* values were assigned a value of 10^4^ for illustrative purposes.

A number of well-characterized RBE trends in the literature are qualitatively reproduced by our model, particularly as it relates to the response of cells with vastly differing radiosensitivities. To illustrate this, we show our model’s predictions of the LET response for the data published by Weyrather *et al* (1999), with cells ranging in sensitivity from D_10%_ = (7.8 ± 0.7) Gy to D_10%_ = (1.34 ± 0.07) Gy (figure 3). Our model predicts the overkill effect for high LET values (including that RBE_max_ occurs at different LET values for different cell lines), and that radiosensitive cells tend to have smaller RBE values than radioresistant cells (including RBE values <1 for very radiosensitive cells) (figure 3(A)). Furthermore, it predicts that the behavior of *α_C_* mimics that of the RBE trends (figure 3(B)) while the *β_C_* values tend towards zero for higher LET values (figure 3(C)), resulting in linearized cell survival curves as *α_C_*/ *β_C_* tends towards infinity for higher LET values (figure 3(D)).

### 3.7. Accuracy of our model’s predictions

**Figure 4.**
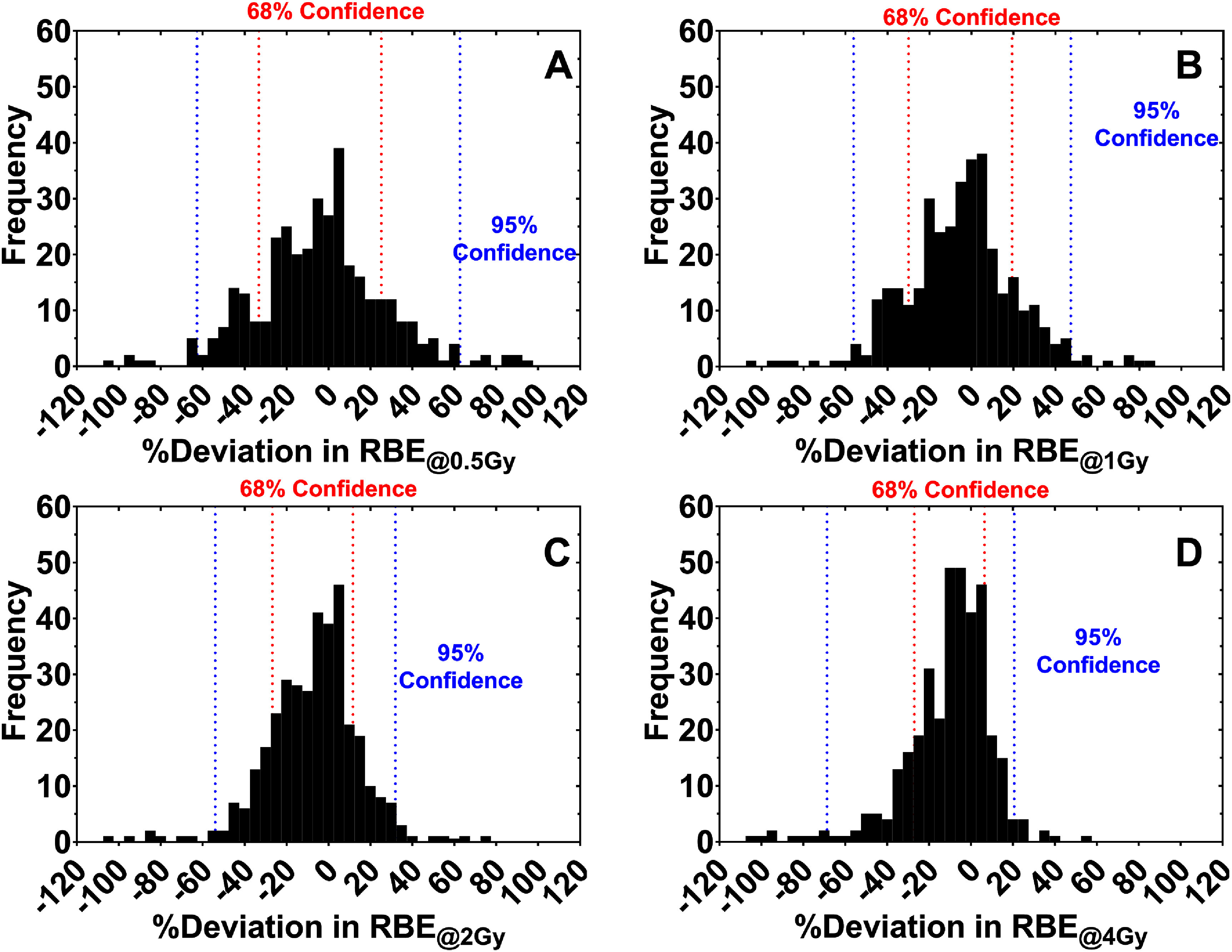
Percent deviations in our model’s predicted RBE values at the (A) 0.5 Gy, (B) 1 Gy, (C) 2 Gy, and (D) 4 Gy dose levels after leave-one-out cross-validation. The red and blue lines show the bootstrapped 68% and 95% confidence intervals.

We used leave-one-out cross-validation to broadly assess the accuracy of our model across the training data and calculated the relative error in the predicted RBE values at the 0.5, 1, 2, and 4 Gy dose levels before bootstrapping the deviations to estimate confidence intervals. The accuracy of our model’s predictions across the whole dataset was on the order of ±20% at the 68.3% confidence level, as shown in figure 4 (see supplementary note S5 for tabulated prediction intervals).

**Figure 5.**
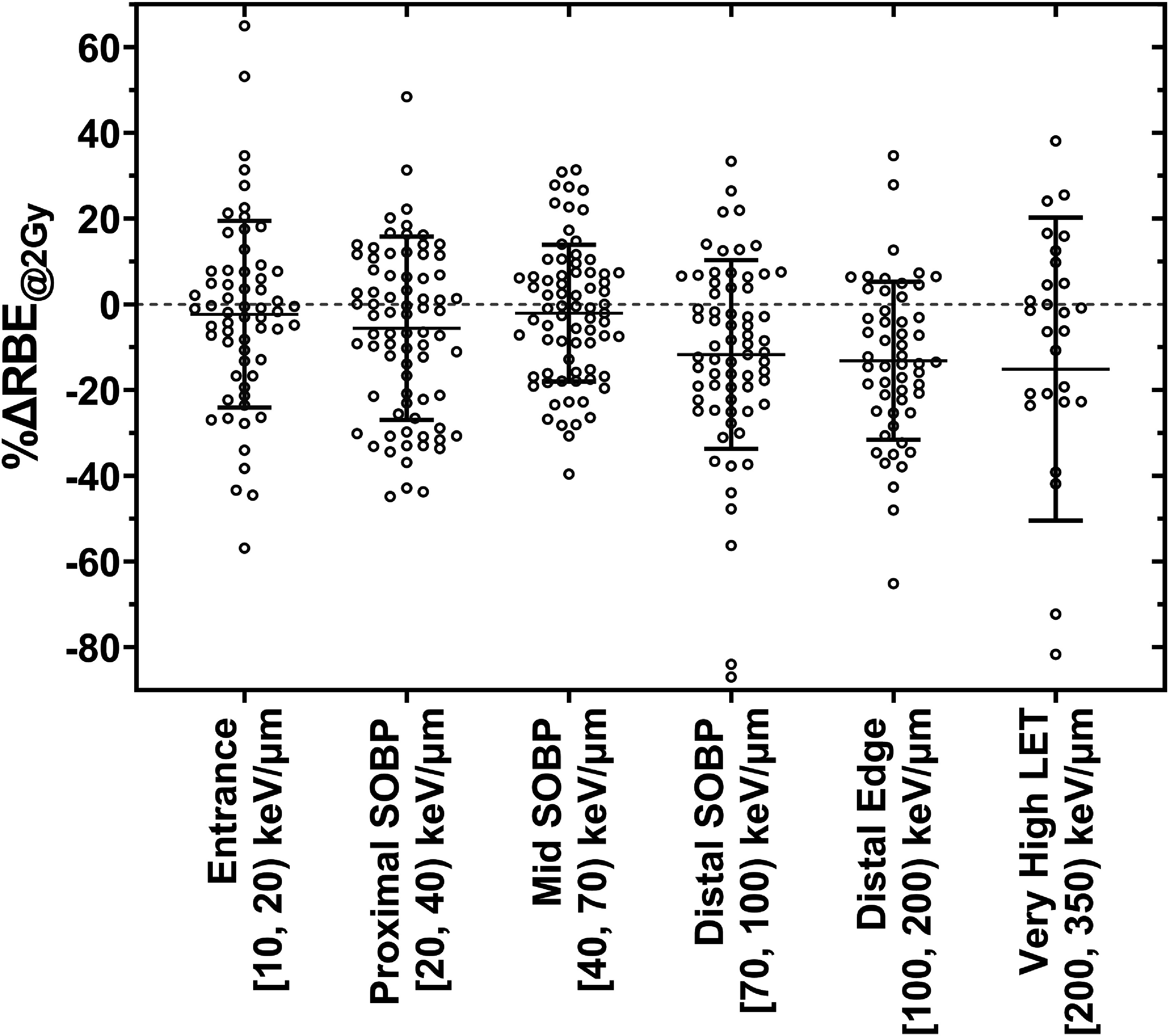
Relative deviations in RBE predicted by our model across the training data for LET regions corresponding approximately to the entrance (LET < 20 keV *μ*m^−1^), the proximal SOBP (20 keV *μ*m^−1^ ⩽ LET < 40 keV *μ*m^−1^), mid SOBP (40 keV *μ*m^−1^ ⩽ LET < 70 keV *μ*m^−1^), distal SOBP (70 keV *μ*m^−1^ ⩽ LET < 100 keV *μ*m^−1^), distal edge (100 keV *μ*m^−1^ ⩽ LET < 200 keV *μ*m^−1^), and very high LET values (LET ⩾ 200 keV *μ*m^−1^). Bars show the mean and standard deviation of the relative deviations indicating any prediction bias (mean) and the prediction accuracy (standard deviation).

To assess how the model’s predictions varied as a function of LET, we further subdivided the data into LET categories approximately corresponding to the LET values found at the entrance (LET *<*20 keV *μ*m^−1^), the proximal SOBP (20 keV *μ*m^−1^ *⩽*LET < 40 keV *μ*m^−1^), mid SOBP (40 keV *μ*m^−1^ *⩽* LET *<*70 keV *μ*m^−1^), distal SOBP (70 keV *μ*m^−1^ *⩽* LET *<*100 keV *μ*m^−1^), distal edge (100 keV *μ*m^−1^ *⩽* LET < 200 keV *μ*m^−1^), and very high LET (LET ⩾ 200 keV *μ*m^−1^) regions. These data (figure 5) show that while the accuracy of our model’s predictions was relatively stable across clinically relevant LET values, it was less accurate for very high LET values. Also, for higher LET values, our model tended to systematically under predict the RBE by about 10%; however, the presence of a small number of datasets that included extraordinarily high RBE values may bias this assessment.

### 3.8. Comparison to other RBE models

Mein *et al* (2020) compared the accuracy of the LEM and MKM for predicting the response of four cell lines exposed to C-ions for LET*_d_* values ranging from 23 keV *μ*m*^−1^* to 107 keV *μ*m*^−1^*. To quantify the accuracy of the predictions of these models, they calculated the mean absolute relative deviations in the predicted versus measured survival curves via the endpoints D_10%_ and D_50%_, and also the RBE at the 1, 2, and 4 Gy dose levels. Their results are summarized in table 4 alongside the same analyses done with our model’s predictions of their data. Here, we used the predictions made for Mein’s data during our leave-one-out cross-validation described above to better quantify the predictive power of our model. Generally, these results show that our model offers comparable accuracy to both the LEM and MKM models.

**Table 4.** Relative accuracy of RBE model predictions using the LEM1, LEM4, and mMKM models published by Mein *et al* (2020) for their cell survival data (A549, H460, B16, and RENCA cells exposed to six C-ion LET_*d*_ values ranging from 23 keV *μ*m^−1^ to 107 keV *μ*m^−1^) alongside our model’s performance for the same data.

Model	$\overline {\% \Delta {{\text{D}}_{10\% }}} $	$\overline {\% \Delta {{\text{D}}_{50\% }}} $	$\overline {\% \Delta {\text{RB}}{{\text{E}}_{1\,{\text{Gy}}}}} $	$\overline {\% \Delta {\text{RB}}{{\text{E}}_{2\,{\text{Gy}}}}} $	$\overline {\% \Delta {\text{RB}}{{\text{E}}_{4\,{\text{Gy}}}}} $
LEM1	10.1 ± 3.1	22.4 ± 11.8	20.2 ± 10.8	14.6 ± 5.5	8.6 ± 0.3
LEM4	20.6 ± 3.1	18.6 ± 7.8	13.3 ± 4.6	12.5 ± 3.2	12.6 ± 0.6
mMKM	4.5 ± 1.1	11.9 ± 10.5	7.9 ± 6.0	4.6 ± 3.2	2.8 ± 0.6
Our model	12.6 ± 3.9	18.1 ± 4.0	14.6 ± 2.6	10.0 ± 2.0	7.2 ± 1.4

## 4. Discussion

Our previous work showed that the strong linear correlations between proton and photon radiosensitivity can be leveraged to create an accurate empirical model of proton RBE (Flint *et al* 2022), and here we show the same is true for C-ion RBE. In the context of protons, we previously noted the trends predict a non-linear relationship between ion RBE and photon radiosensitivity, resulting from the linear relationship between radiosensitivity to protons versus photons. This generally implies that radioresistant cells will have larger RBE values, radiosensitive cells will have smaller RBE values, and we will occasionally see RBE values <1 for very radiosensitive cells (Flint *et al* 2022). Here, we show that this general trend remains true for our C-ion model as well. This suggests that our formalism—characterizing the LET dependence of the linear correlations between radiosensitivity to ions versus photons—may be generally applicable across ion species since the underlying trends do not seem to depend on ion type.

However, a limitation of our previous work was that we could not compare the accuracy of our approach to that of mechanistic approaches such as LEM or MKM because the input parameters necessary to perform the calculations with those models are not readily available, leaving a lingering question as to the relative strengths and weaknesses of mechanistic versus empirical approaches in RBE modeling (Flint *et al* 2022). In the current work, however, thanks to the findings reported by Mein *et al* (2020), we were able to make this direct comparison for four cell lines across six LET values and show that the accuracy of our empirical approach is indeed comparable to the much more sophisticated mechanistic approaches. We believe that this is a great step forward for empirical RBE modeling, because to our knowledge this is the first time such a comparison has been made in the context of C-ions, and remarkably, this comparison shows that we do not suffer a significant loss of accuracy in the absence of any sophisticated mechanistic basis for our mathematical description of the C-ion data.

Moreover, our approach is substantially less complex than any of the mechanistic approaches. Our model incorporates only four free (fit) parameters into its framework, and the user must specify only three input parameters—the reference *α_X_* and *β_X_* values (which are required by all models) and LET*_d_* as the beam quality specifier. Our model simply requires the evaluation of an algebraic expression, which can be done with very little computational expense, and, which we previously showed for protons, can be easily incorporated into a clinical treatment planning workflow (Flint *et al* 2022). To illustrate the simplicity of our approach, we have implemented our model into an Excel spreadsheet and a MATLAB function which we have included as supplementary material. This simplicity uniquely places our empirical approach in a position where RBE-weighted doses can be rapidly calculated and re-calculated across the voxels of a treatment planning CT, because the only input parameters needed are those already known (the reference *α_X_* and *β_X_* values) or those that can be scored rapidly by using fast Monte Carlo techniques and track-repeating algorithms (Yepes *et al* 2009, Wang *et al* 2019) (the LET*_d_*).

An important benefit of this simplified approach may be seen in clinical contexts where the lack of necessary input parameters and the computational expense of performing calculations with the existing mechanistic models mean that RBE predictions are made using the responses of only a few reference cell lines, and often requires that RBE values be recalculated and used via lookup tables (Scholz *et al* 1997, Krämer and Scholz 2000, Karger and Peschke 2017). Although using recalculated RBE values for reference cell lines of a given disease site may be a pragmatic solution to overcome this limitation, we recently showed that, even among cells of the same histologic type (which would receive the same dose prescription in a clinical setting), great variability exists in intrinsic C-ion radiosensitivity and RBE, and that the variability in RBE increases with C-ion LET (Flint *et al* 2021b). Thus, in an ideal scenario (and hopefully in the future of C-ion therapy), patient-specific radiosensitivity could be predicted in addition to RBE to account for this variability. But because neither empirical nor mechanistic RBE models attempt to address the issue of inter-tumor heterogeneity in intrinsic radiosensitivity (although some work has been done to include corrections in the MKM to account for intra-tumor heterogeneity (Inaniwa *et al* 2024)), a significant limitation of RBE modeling, whether empirical or mechanistic, is that photon survival data are needed as input parameters, and this type of data is rarely available in clinical settings. However, a great deal of research is currently underway to develop predictive models of intrinsic cell radiosensitivity (Chavaudra *et al* 2004, Chistiakov *et al* 2008, Torres-Roca, 2012, Borras-Fresneda *et al* 2016), and empirical approaches such as ours need only this radiosensitivity information as their cell-specific input parameters (*α_X_* and *β_X_*). Thus, empirical models such as ours may be well-positioned to incorporate our improving understanding of the factors that drive intrinsic radiosensitivity into our estimation of RBE in clinical scenarios, whereas the mechanistic approaches may still be too cumbersome to incorporate this radiosensitivity information within a clinically realistic timeframe.

That said, one substantial limitation of our work is its purely empirical nature; our formalism does not offer much insight into the physical and biological factors governing the trends in the data—it merely characterizes them. Thus, although our model may offer pragmatic advantages in terms of ease of use, one cannot dismiss the much more informative nature of mechanistic modeling approaches towards understanding why the data trend the way they do. Although we believe our approach can serve as a pragmatic tool to use when rapid, accurate RBE estimates are required, or simply in scenarios where the input parameters for the mechanistic models cannot be obtained, we caution against placing much emphasis on understanding the particular functions themselves that our model uses. This sentiment arises largely due to the great noise in the biological training data and how this noise affects our ability to characterize the underlying trends unambiguously—the distance metrics used in model fitting (sum-of-squares distances) are greatly influenced by outlying data, and thus small changes to how the training data are curated, or how one’s distance metric is defined, can result in a different choice of functions that ‘best fit’ the data. It is therefore difficult to assign much meaning to the functions themselves, aside from saying that they fit the subset of data that were assessed as well as those data can be fit.

Indeed, the functions we found that best describe the C-ion data align only qualitatively with the functions we previously found to describe the proton response, e.g. here we modeled the response of the slope of the linear correlations with an inverse linear function, whereas in our proton model, we used a negative exponential function to describe the slope’s LET dependence—both describing a slope that decays with LET, albeit by slightly different mathematical prescriptions (Flint *et al* 2022). Although one might expect these trends to be described by functions of the same form across ion types, it should not be alarming that this is not the case: it could suggest simply that, owing to the noise in the data, the specific choice of functions describing the underlying trends does not strongly influence the quality of the resulting model, or that a more complex beam quality specifier such as the *Q* factor reported by Luhr *et al* (2017) may be needed to reconcile the response between ion types. But, given that the basic trends underpinning our model—the correlation between ion and photon radiosensitivity—holds for both C-ions and protons, this trend likely holds for all clinically relevant ions, and each ion’s response can simply be modeled independently by using LET*_d_* as the beam quality specifier. Notably, the data in the PIDE database contain both monoenergetic beams and mixed fields, but the LET*_d_* values are specified for each condition, implying that in the context of our model, LET*_d_* alone may be sufficient to characterize the beam quality, even for mixed fields. This is a great advantage of our approach over the LEM or MKM models, where much more sophisticated beam quality specifies are needed to account for the presence of different ion species in mixed fields (Stewart *et al* 2018).

Nevertheless, the observation that the linear relationship between ion and photon radiosensitivity seems to hold across radiation types may merit some explanation. This proportionality might be explained in part when we consider how the induction of lethal DNA lesions relates to, for example, the quantity D_10%_. Under the framework of the Curtis Lethal-Potentially Lethal (LPL) model (Curtis 1986), a given dose of radiation will induce some number of DNA lesions, some of which are lethal and result in cell death, and others are only potentially lethal if not repaired. But D_10%_ occurs well beyond the repair shoulder (where the repair of potentially lethal lesions is most relevant) and so cell death in the vicinity of D_10%_ is dominated by the induction of irreparable, lethal lesions. Consequently, D_10%_ can be understood as the dose resulting in a 90% probability of inducing at least one lethal lesion. Because the number of lesions induced by a given radiation dose follows a Poisson distribution (Hawkins 1994), D_10%_ occurs when the number of lethal lesions per cell, *η*, has an expectation value of ∼2.3. But if we assume, as in the LPL model, that in the vicinity of D_10%_ the expected number of lethal lesions induced is proportional to dose (Curtis 1986), then D_10%,*C*_, and D_10%,*X*_ are both proportional to the same quantity, *η* ≈ 2.3, and therefore they are both proportional to one another. Similar arguments can be made for the other radiosensitivity parameters examined.

This explanation may break down for very high LET values, where the distribution of lethal lesions across a population of cells may no longer strictly follow a Poisson distribution owing to inhomogeneities in the microscopic dose distributions for high LET ions, which in turn may explain the relatively poorer performance of our model for very high LET values (figure 5). However, given the strong linear correlations between D_10%,*X*_ and D_10%,*C*_ directly observed for up to at least 77 keV *μ*m^−1^ (*R* = 0.9592) by Suzuki *et al* (2000), and the relatively stable performance of our model for up to ∼200 keV *μ*m^−1^ (figure 5), this breakdown likely does not occur until LET values far higher than the clinically relevant range.

The relatively poorer performance of our model for very high LET values may also simply be explained by the relative sparsity of high LET data within the training dataset, resulting in the global fit of our model being weighted towards where there are the most published data: the regions corresponding to the entrance or middle of the SOBP in C-ion beams. However, this limitation (that deviations between the model predictions and the experimental data tend to be relatively large for very high LET values) is shared among mechanistic and empirical models alike. For example, in the case of the MKM, this has led to modifications to the model to try account for the stochastic nature of radiation deposition in cell nuclei in the high LET (low fluence) regime, which has been shown to improve the MKM’s predictions (Sato and Furusawa 2012). It may therefore be likely that more complex mathematical functions are needed in empirical approaches to precisely characterize cell response to increasing LET values as the response transitions from being driven by increased cell inactivation to being driven by decreasing particle fluence. However, until more high-LET data become available, we remain limited in how many free parameters we can justify including in such an approach, which largely precludes the use of functions considerably more complex than those we investigated. In any case, highly accurate mapping of RBE for LET values far beyond RBE_max_ may not be of great clinical significance in the context of C-ion therapy as we are unlikely to encounter clinically significant regions with LET*_d_* values considerably higher than 200 keV *μ*m^−1^.

## 5. Conclusions

Our work confirms that C-ions radiosensitivity correlates with photon radiosensitivity and that this relation can be used to model cell radiosensitivity, RBE, and predict the survival curves of cells exposed to C-ions based on their response to photons. Our empirical model contains only four free parameters, requires only three input parameters, is less computationally expensive to use, and makes predictions with comparable accuracy to the LEM and MKM models over a clinically relevant range of C-ion LET values. Our model can be used to predict C-ion RBE values in cases where the input parameters for LEM or MKM are not available, where the rapidity of the calculation is paramount, or where the full implementation of the LEM or MKM is not warranted, without sacrificing prediction accuracy.

## Data Availability

All data that support the findings of this study are included within the article (and any supplementary information files).
